# A case-control study of phosphodiesterase-5 inhibitor use and Alzheimer’s disease and related dementias among male and female patients aged 65 years and older supporting the need for a phase III clinical trial

**DOI:** 10.1371/journal.pone.0292863

**Published:** 2023-10-18

**Authors:** David S. Henry, Richard G. Pellegrino

**Affiliations:** Baptist Health Center for Clinical Research, Little Rock, Arkansas, United States of America; Weizmann Institute of Science, ISRAEL

## Abstract

**Background:**

Phosphodiesterase-5 inhibitors (PDE5i) have been evaluated as a novel treatment for Alzheimer’s disease and related dementias (ADRD), but two recent cohort studies have offered opposing conclusions.

**Methods:**

We performed an unmatched case-control study using electronic medical records from a large healthcare system to evaluate the association of PDE5i use and ADRD in patients ≥65 years old.

**Results:**

Odds of PDE5i exposure were 64.2%, 55.7%, and 54.0% lower in patients with ADRD than controls among populations with erectile dysfunction, benign prostatic hyperplasia, and pulmonary hypertension, respectively. We observed odds ratios less than unity among males and females and with exposure to the PDE5i sildenafil (Viagra®) and tadalafil (Cialis®). We also evaluated the odds of exposure to two other common treatments for pulmonary hypertension: endothelin receptor antagonists (ERA) and calcium channel blockers (CCB). The odds of ERA exposure were 63.2% lower, but the odds of CCB exposure were 30.7% higher, in patients with ADRD than controls among the population with pulmonary hypertension.

**Conclusions:**

Our results reconcile the opposing conclusions from the previous observational studies and support further research into using PDE5i for prevention and treatment of ADRD.

## Introduction

There are currently only six FDA-approved therapies to treat Alzheimer’s disease (AD): cholinesterase inhibitors (donepezil, galantamine, and rivastigmine), an n-methyl-D-aspartate receptor antagonist (memantine), and two anti-amyloid antibodies (aducanumab and lecanemab) [1–3]. However, clinical studies of the cholinesterase inhibitors and memantine have demonstrated, at best, only modest statistical benefits in cognition, which may not translate to meaningful benefits for patients and caregivers in daily life [4, 5]. Additionally, these drugs can have numerous dose-dependent side effects, limiting their tolerability [6, 7]. The antibody therapies were approved by the FDA via an expedited pathway based on limited data demonstrating their benefit, which has received extensive criticism [8–13]. Lecanemab later received full approval by the FDA after the randomized, controlled, phase three Clarity AD trial, which demonstrated an average benefit of only 0.45 points versus placebo on the Clinical Dementia Rating–Sum of Boxes scale, which rates dementia from 0 (normal) to 18 (severe dementia), after 18 months among patients with early AD [10, 14, 15]. The magnitude of this benefit may not be clinically perceptible to patients and caregivers [16]. Additionally, side effects were greater in the lecanemab group, including amyloid-related imaging abnormalities (ARIA) with cerebral microhemorrhages or hemosiderin deposits (14.0% versus 7.7%), ARIA with cerebral edema or effusions (12.6% versus 1.7%), infusion-related reactions (26.4% versus 7.4%), and adverse events leading to discontinuation of the trial agent (6.9% versus 2.9%). The internal and external validity of the Clarity AD trial also have been questioned due to possible inclusion bias, unblinding, and variable drop-out rates between the groups [16]. A third monoclonal antibody, donanemab, was rejected from the expedited approval pathway by the FDA due to an insufficient sample size of patients taking the drug for 12 months in Eli Lilly’s phase 2 clinical trial [17]. In response to this criticism, Eli Lilly recently published the results of the randomized, controlled phase 3 TRAILBLAZER-ALZ 2 trial evaluating the efficacy of donanemab, which demonstrated an average slowing of AD progression by 0.67 points on the CDR-SB scale over 76 weeks. This is similar in magnitude to lecanemab in the Clarity AD trial [18]. Similar side effects were demonstrated in TRAILBLAZER-ALZ 2 as in the Clarity AD trial. Furthermore, the annual cost of the FDA-approved monoclonal antibodies ($28,000 for aducanumab and $56,000 for lecanemab) is prohibitive for many patients, and there is no reason to believe that donanemab will be significantly more affordable [9, 12]. Consequently, there is a pressing need for the expeditious development, testing, and approval of affordable and tolerable drugs to treat AD that can show meaningful benefits to patients and caregivers by preventing or slowing progression of the disease.

Recently, there has been a push for research into repurposing existing FDA-approved drugs to treat and/or prevent AD, because the safety profiles of the drugs have already been established [19, 20]. The phosphodiesterase inhibitors are one such class of drugs. The phosphodiesterase-5 inhibitors (PDE5i) sildenafil and tadalafil belong to a class of medications first developed as antihypertensive and antianginal medications due to their vasodilatory properties. However, they have been approved by the FDA for several other indications: erectile dysfunction (ED), benign prostatic hyperplasia (BPH), and pulmonary hypertension (pHTN) [21].

For more than two decades, pre-clinical functional studies of PDE5i in murine models have demonstrated promising benefits regarding learning and memory. These studies have provided support for a class effect for PDE5i in offering benefits in multiple cognitive domains for both males and females in healthy and diseased populations [22–34]. However, the historical discordance between the results of preclinical models used in AD research and the results of human clinical trials, likely a result of differences in regional distributions of protein subtypes and isoforms in the brain between the various models and humans, highlights the necessity of human clinical studies [19, 35]. Importantly, however, the expression of PDE5i protein in neurons and cerebral blood vessels has been demonstrated [36].

Several small human studies have demonstrated mixed results regarding the effect of PDE5i on cognition, often with beneficial effects seen in cognitive processes but usually not in clinically significant outcomes. In a study of ten healthy male subjects, a single dose of sildenafil (100 mg) produced differences in event-related brain potentials during tests of attention and word recognition between the sildenafil and placebo groups, although no effect on cognitive outcomes were observed in these studies [37]. A second study demonstrated statistically significant improvement with a single dose of sildenafil (100 mg) in only 1 of 37 parameters during cognitive testing among six healthy male subjects [38]. A third study demonstrated decreases in fractional amplitude of low frequency fluctuation (fALFF) in the right hippocampus after a single dose of sildenafil (50 mg) among 10 patients with early AD [39]. However, a fourth study failed to demonstrate improvement in cognitive tasks involving memory, planning, attention, or motor speed associated with single doses of 10 or 20 mg of the PDE5i vardenafil among 18 healthy university students [40].

Longer-term administration of PDE5i may demonstrate more benefits on cognitive outcomes in humans. Among 25 patients with ED and mild cognitive impairment (MCI), eight weeks of tadalafil (5 mg daily) increased Montreal Cognitive Assessment (MoCA) scores on average 2.88 points from baseline, although no placebo group was included in this study [41]. While suggestive, the significance of this result is unclear, given the small sample sizes.

Recently, two large observational studies used data from insurance claims databases to evaluate the association between chronic PDE5i use and Alzheimer’s disease and arrived at diametrically opposed conclusions [42, 43]. In late 2021, Fang *et al*., after identifying sildenafil as a candidate drug to treat Alzheimer’s disease by *in silico* methods, performed a retrospective cohort study in which they observed lower hazard ratios for sildenafil compared to a propensity score-matched control population. They also found a protective effect of sildenafil compared to several other commonly prescribed medications, and the protective effect of sildenafil on AD risk was consistent across subgroups of patients with five common chronic diseases [42]. Their research supported moving sildenafil forward in the AD drug development pipeline.

In 2022, Desai *et al*. performed a similar retrospective cohort study. However, they limited their study population to patients with pulmonary hypertension (pHTN), included patients taking both sildenafil and tadalafil, expanded the outcome to include Alzheimer’s disease-related dementias (ADRD), and used patients taking endothelin receptor antagonists as the control group. With these new conditions, they found that the hazard ratio between the PDE5i exposure group and ERA exposure group was not significantly different from unity [43]. Based on their results, they advocated against further testing of PDE5i for the treatment of ADRD.

The present case-control study was designed to reconcile the opposing conclusions of these recent studies by Fang *et al*. and Desai *et al*. in order to determine whether further research into the efficacy and effectiveness of PDE5i in preventing and/or treating ADRD is warranted.

## Methods

We used an unmatched case-control design to evaluate the association between history of PDE5i (sildenafil and tadalafil) use and ADRD. All patients included in the study were identified from the Epic electronic medical record database of Baptist Health, a large integrated health system in Arkansas, USA, which includes medical records for more than 1.4 million patients across the health system. All patients included in this study had at least one outpatient provider encounter between 1 January 2018 and 31 December 2022. Due to limitations in the data analysis platform we used, patients were at least 65 years old at the time of data tabulation (January 2023 to March 2023), which accounts for minor variability in the aggregate patient counts. Additionally, inclusion in this study was restricted to patients with at least one FDA-approved chronic indication for PDE5i (ED, BPH, or pHTN), and throughout the study, we restricted each analysis by PDE5i indication in order to minimize confounding by indication. WCG IRB issued a waiver of HIPAA authorization for this study (Protocol No.: II-001). Only aggregate data were obtained during this study, and no protected health information was used.

When evaluating patients with BPH, an indication that had not been examined specifically before, we excluded patients with comorbid ED or pHTN. Unlike ED and pHTN, tadalafil is the only PDE5i which is FDA-approved for BPH. BPH is not an FDA-approved indication for sildenafil. Additionally, the recommended dosages of tadalafil for BPH (5 mg daily) are lower than for pHTN (20–40 mg daily). Furthermore, unlike ED, for which patients may take daily or as needed doses of tadalafil, patients with BPH must take tadalafil daily. By excluding patients with comorbid ED or pHTN, we were able to evaluate the association of daily low-dose tadalafil use, unadulterated by high doses or “as needed” regimens, and ADRD [44, 45]. Aggregate patient counts and measures of centrality and dispersion were tabulated using Epic’s SlicerDicer tool. No patient-level data, including protected health information, were accessed or used in this study. SlicerDicer queries for drug exposures included all formulations with the drug of interest, including combination drugs. ICD-10 codes and SNOMED descriptors for diseases used in SlicerDicer queries are included in S1 Table and were input into both the diagnosis and the medical history fields with a logical “or” operator. Two-tailed tests were performed using the epitools package in R (version 4.3.0), and an α-value of 0.05 was used as the *a priori* cutoff for significance. All patient frequencies used in the calculation of descriptive statistics are included in S2, S3 Tables.

Because we used the inclusive outcome of ADRD, similar to Desai *et al*., we repeated the previous analysis using the more selective outcome of AD, exclusive of related dementias, similar to Fang *et al*. [42, 43]. When evaluating AD without related dementias a, we excluded patients with the related dementias from the control group.

Because Desai *et al*. used endothelin receptor antagonists (ERA; ambrisentan, bosentan, and macitentan) for the control group in their study and found hazard ratios that did not differ from unity, we also evaluated the association between history of ERA use and ADRD [43]. We chose to evaluate ERA use as a separate exposure instead of using it to identify the unexposed group because it spoke more directly to our main question: Are the odds of exposure to PDE5i, ERA and/or calcium channel blockers (CCB) less than unity among patients with ADRD or AD when compared to similar patients with no exposure to these medications? Comparisons of the odds ratios between the different drug classes is a secondary question, which also is evaluated by our chosen experimental design.

While Fang *et al*. found a greater reduction in ADRD risk among males on sildenafil than females on sildenafil, they did not restrict by indication and consequently observed substantially higher average sildenafil doses among males than among females [42]. Desai *et al*. did not compare males and females with pHTN treated with PDE5i to untreated controls [43]. We therefore evaluated the association of ADRD with PDE5i and ERA use versus no PDE5i or ERA use, respectively, among males and females with pHTN as separate analyses.

We also compared the effect of sildenafil and tadalafil in patients with ED and pHTN. We could not make this comparison in BPH, as only tadalafil is FDA-approved for treatment of BPH [44, 45]. Fang *et al*. only evaluated the effect of sildenafil on AD risk, while Desai *et al*. evaluated the effect of sildenafil and/or tadalafil on ADRD risk but did not conduct separate sub-analyses for each drug. In this evaluation, we compared patients with a history of sildenafil but not tadalafil use to patients with a history of tadalafil but not sildenafil use in subjects with ED and pHTN. Similarly, we evaluated the association of the use of each ERA and history of ADRD among patients with pHTN. However, due to small sample sizes, we included patients with a history of using more than a single drug in the ERA class.

Fang *et al*. evaluated four additional comparator drugs: the CCB diltiazem, the angiotensin receptor blocker losartan, the sulfonylurea glimepiride, and metformin. These evaluations were repeated in patient populations with coronary artery disease, systemic hypertension, and type 2 diabetes mellitus. However, patients in these analyses were not restricted by PDE5i indication [42]. Because CCB and PDE5i share an indication, pHTN, we also evaluated CCB (amlodipine, diltiazem, and nifedipine) as a comparator group among patients with pHTN [46]. Finally, because neither Fang *et al*. nor Desai *et al*. evaluated the effect of age, we evaluated whether age is an effect modifier for the association of PDE5i use and ADRD in ED, BPH, and pHTN. These analyses will serve to inform the design of future retrospective and prospective studies.

## Results

We began by evaluating whether the odds of PDE5i exposure is different for patients with ADRD versus controls without ADRD among populations with each chronic FDA-approved indication for PDE5i: ED, BPH, and pHTN. Demographic and clinical characteristics of all patients over 65, stratified by exposure and outcome, are summarized in Table 1. For ED, BPH, and pHTN, the odds of exposure to PDE5i are significantly less among patients with ADRD versus controls without ADRD. The same is true for ERA exposure in pHTN (Table 2).

**Table 1:** Characteristics of patients with history of Alzheimer’s disease and related dementias (ADRD) and controls stratified by drug exposure.

Characteristic^1^	AD	No ADRD			
	All	All	PDE5i only	PDE5i + ERA	ERA only
	n = 17,565	n = 227,530	n = 9,942	n = 111	n = 56
**Demographics**					
Age, mean (SD)	83 (8)	75 (7)	72 (6)	75 (6)	75 (8)
Male	6,942 (39.5)	98,119 (43.1)	9,686 (97.4)	35 (31.5)	12 (21.4)
White	14,818 (84.4)	185,049 (81.3)	8,227 (82.7)	88 (79.3)	46 (82.1)
**Dementia risk factors**					
Smoking history					
Current smoker	1,468 (8.4)	21,863 (9.6)	1,133 (11.4)	2 (1.8)	2 (3.6)
Never smoker	9,401 (53.5)	101,485 (44.6)	4,581 (46.1)	54 (48.7)	34 (60.7)
BMI ≥ 25	11,604 (66.1)	142,298 (62.5)	8,774 (88.3)	93 (83.8)	43 (76.8)
Hypertension	15,349 (87.4)	150,327 (66.1)	8,518 (85.7)	103 (92.8)	49 (87.5)
Diabetes	6,373 (36.3)	57,373 (25.2)	3,345 (33.7)	53 (47.7)	23 (41.1)
Coronary artery disease	7,229 (41.2)	55,880 (24.6)	3,635 (36.6)	60 (54.1)	25 (44.6)
Ischemic stroke	4,762 (27.1)	19,309 (8.5)	793 (8.0)	16 (14.4)	7 (12.5)
Atrial fibrillation	5,671 (32.3)	32,731 (14.4)	1,874 (18.8)	62 (55.9)	16 (28.6)
Venous or pulmonary embolism	1,579 (9.0)	9,445 (4.2)	532 (5.3)	21 (18.9)	13 (23.2)
**Indications for PDE5i or ERA**					
ED^2^	322 (4.6)	6,645 (6.8)	3,976 (41.0)	0	0
BPH only^2^	2,354 (33.9)	18,283 (18.6)	1,603 (16.5)	0	4 (33.3)
pHTN	1,125 (6.4)	6,694 (2.9)	717 (7.2)	105 (94.6)	41 (73.2)

^1^ Reported as n (%), unless otherwise specified

^2^ Proportion reported as % of males

Additional abbreviations: PDE5i, phosphodiesterase-5 inhibitor; ERA, endothelin receptor antagonist; BMI, body mass index; ED, erectile dysfunction; BPH, benign prostatic hyperplasia; pHTN pulmonary hypertension.

**Table 2:** Odds ratios for history of phosphodiesterase-5 inhibitor (PDE5i) and/or endothelin receptor antagonist (ERA) use in patients with Alzheimer’s disease and related dementias (ADRD) versus controls without ADRD, stratified by phosphodiesterase-5 inhibitor (PDE5i) or endothelin receptor antagonist (ERA) indication: Erectile dysfunction (ED), benign prostatic hyperplasia (BPH), and pulmonary hypertension (pHTN). Fisher’s exact test for significance of odds ratios.

Analysis	Indication	Odds ratio (95% CI)	p-value
PDE5i v. no PDE5i	ED	0.358 (0.283–0.452)	< 0.001
	BPH only	0.443 (0.357–0.544)	< 0.001
	pHTN	0.460 (0.353–0.590)	< 0.001
ERA v. no ERA	pHTN	0.368 (0.173–0.682)	0.001

Next, we performed a similar analysis evaluating AD instead of ADRD. Demographic and clinical characteristics for patients with the diagnosis of AD and those with ADRD are summarized in Table 3. For each FDA-approved indication of PDE5i, the odds of exposure to PDE5i is significantly less among patients with AD versus those without ADRD. Again, the same is true for ERA exposure in pHTN. Furthermore, the odds ratios for patients with ED, BPH, and pHTN exposed to PDE5i or ERA, using the AD as the outcome are not statistically different from the odds ratios with the more inclusive outcome of ADRD (Table 4).

**Table 3:** Characteristics of patients with Alzheimer’s disease (AD) and Alzheimer’s disease and related dementias (ADRD).

Characteristic^1^	AD	ADRD
	n = 7,198	n = 17,565
**Demographics**		
Age, mean (SD)	83 (8)	83 (8)
Male	2,611 (36.3)	6,942 (39.5)
White	6,173 (85.8)	14,818 (84.4)
**Dementia risk factors**		
Smoking history		
Current smoker	477 (6.6)	1,468 (8.4)
Never smoker	4,081 (56.7)	9,401 (53.5)
BMI ≥ 25	4,593 (63.8)	11,604 (66.1)
Hypertension	6,202 (86.2)	15,349 (87.4)
Diabetes	2,336 (32.5)	6,373 (36.3)
Coronary artery disease	2,624 (36.5)	7,229 (41.2)
Ischemic stroke	1,596 (22.2)	4,762 (27.1)
Atrial fibrillation	2,081 (28.9)	5,671 (32.3)
Venous or pulmonary embolism	479 (6.7)	1,579 (9.0)
**Indications for PDE5i or ERA**		
ED^2^	112 (4.2)	322 (4.6)
BPH only^2^	928 (35.5)	2,354 (33.9)
pHTN	337 (4.7)	1,125 (6.4)

^1^ Reported as n (%), unless otherwise specified

^2^ Proportion reported as % of males

Additional abbreviations: PDE5i, phosphodiesterase-5 inhibitor; ERA, endothelin receptor antagonist; BMI, body mass index; ED, erectile dysfunction; BPH, benign prostatic hyperplasia; pHTN pulmonary hypertension.

**Table 4:** Odds ratios for history of phosphodiesterase-5 inhibitor (PDE5i) and/or endothelin receptor antagonist (ERA) use in patients with Alzheimer’s disease (AD) versus controls without AD or a related dementia (ADRD), stratified by phosphodiesterase-5 inhibitor (PDE5i) or endothelin receptor antagonist (ERA) indication: Erectile dysfunction (ED), benign prostatic hyperplasia (BPH), and pulmonary hypertension (pHTN). Fisher’s exact test for significance of AD odds ratios. Odds ratios for ADRD added from Table 2 for comparison.

Analysis	Indication	AD Odds ratio (95% CI)	p-value	ADRD
PDE5i v. no PDE5i	ED	0.340 (0.231–0.495)	< 0.001	0.358
	BPH only	0.557 (0.408–0.741)	< 0.001	0.443
	pHTN	0.544 (0.348–0.811)	0.003	0.460
ERA v. no ERA	pHTN	0.151 (0.007–0.663)	0.010	0.368

We also evaluated whether sex functions as an effect modifier for the association of PDE5i and ERA with ADRD. Because pHTN is the only indication for PDE5i and ERA among both males and females, we restricted the study population to patients with this. A comparison of demographic and clinical characteristics among patients with pHTN stratified by ADRD status is summarized in Table 5. In Table 6, we demonstrate that the odds of PDE5i use were significantly reduced among males and females with ADRD versus controls without ADRD. Similarly, the odds ratio for ERA use was significantly lower than unity for females. Among males, however, the odds ratio for ERA use was similar to PDE5i use in magnitude but did not reach statistical significance. Notably, the paucity of males treated with ERA for pHTN limited statistical power. We were unsure of the extent to which males with pHTN were treated with PDE5i as needed to treat their ED instead of scheduled daily doses to treat their pHTN or on lower doses to treat symptoms of BPH. Consequently, we excluded males with ED and/or BPH and observed a similar odds ratio for PDE5i exposure (OR = 0.437, 95% CI: 0.232–0.752, p = 0.002).

**Table 5:** Characteristics of patients with pulmonary hypertension and history of Alzheimer’s disease and related dementias (ADRD) and controls, stratified by drug exposure.

Characteristic^1^	AD	No ADRD			
	All	All	PDE5i only	PDE5i + ERA	ERA only
	n = 1,125	n = 6,694	n = 717	n = 105	n = 41
**Demographics**					
Age, mean (SD)	83 (8)	78 (8)	75 (7)	75 (6)	75 (8)
Male	425 (37.8)	2,721 (40.6)	515 (71.8)	34 (32.4)	6 (14.6)
White	929 (82.6)	5,576 (83.3)	585 (81.9)	83 (79.0)	35 (85.4)
**Dementia risk factors**					
Smoking history					
Current smoker	80 (7.1)	561 (8.4)	59 (8.2)	2 (1.9)	2 (4.8)
Never smoker	538 (47.8)	3,125 (46.7)	301 (42.0)	40 (47.6)	24 (58.5)
BMI ≥ 25	876 (77.9)	5,587 (83.5)	632 (88.1)	90 (85.7)	32 (78.0)
Hypertension	1,071 (95.2)	6,304 (94.2)	672 (93.7)	97 (92.4)	36 (87.8)
Diabetes	536 (47.6)	3,009 (45.0)	329 (45.9)	51 (48.6)	15 (36.6)
Coronary artery disease	787 (70.0)	4,237 (63.3)	484 (67.5)	59 (56.2)	22 (53.7)
Ischemic stroke	380 (33.8)	1,235 (18.4)	113 (15.8)	16 (15.2)	6 (14.6)
Atrial fibrillation	741 (65.9)	3,625 (54.2)	407 (56.8)	60 (57.1)	15 (36.6)
Venous or pulmonary embolism	191 (17.0)	941 (14.1)	98 (13.7)	20 (19.0)	11 (26.8)

^1^ Reported as n (%), unless otherwise specified

^2^ Proportion reported as % of males

Additional abbreviations: PDE5i, phosphodiesterase-5 inhibitor; ERA, endothelin receptor antagonist; BMI, body mass index.

**Table 6:** Odds ratios among patients with pulmonary hypertension for history of phosphodiesterase-5 inhibitor (PDE5i) and/or endothelin receptor antagonist (ERA) use in patients with Alzheimer’s disease and related dementias (ADRD) versus controls without ADRD, stratified by legal sex. Fisher’s exact test for significance of odds ratios.

Analysis	Legal sex	Odds ratio (95% CI)	p-value
PDE5i v. no PDE5i	Male	0.413 (0.290–0.572)	< 0.001
	Female	0.567 (0.373–0.830)	0.003
ERA v. no ERA	Male	0.500 (0.116–1.386)	0.264
	Female	0.323 (0.125–0.678)	0.002

Due to the differences in binding affinities of sildenafil and tadalafil to various ligands, we compared the odds ratios for these drugs individually among patients with ED, BPH, and pHTN [19]. The odds of sildenafil use and tadalafil use were lower for patients with ADRD than without ADRD for each indication, and we observed no significant differences between the two drugs for both ED and pHTN, which are the only chronic conditions for which both drugs are FDA-approved (Table 7). We also evaluated each ERA. The odd ratios for macitentan and ambrisentan were statistically less than unity (Table 8).

**Table 7:** Odds ratios comparing history of sildenafil or tadalafil use between patients with Alzheimer’s disease and related dementias (ADRD) versus controls without ADRD, stratified by drug indication: Erectile dysfunction (ED) and/or pulmonary hypertension (pHTN). Fisher’s exact test for significance of odds ratios.

Indication	Analysis	Odds ratio (95% CI)	p-value
ED	Sildenafil only	0.372 (0.285–0.481)	< 0.001
	Tadalafil only	0.323 (0.205–0.484)	< 0.001
pHTN	Sildenafil only	0.469 (0.350–0.617)	< 0.001
	Tadalafil only	0.479 (0.224–0.897)	0.025

**Table 8:** Odds ratios among patients with pulmonary hypertension for history of endothelin receptor antagonist use in patients with Alzheimer’s disease and related dementias (ADRD) versus controls without ADRD. Fisher’s exact test for significance of odds ratios.

Analysis	Odds ratio (95% CI)	p-value
Macitentan v. no macitentan	0.338 (0.131–0.708)	0.004
Bosentan v. no bosentan	0.705 (0.103–2.465)	0.758
Ambrisentan v. no ambrisentan	0.188 (0.008–0.856)	0.035

To evaluate whether treatment of the underlying pHTN may be responsible in the decreased odds of PDE5i and ERA use in patients with ADRD, we looked at a third class of medications commonly used to treat pHTN, CCB. Demographic and clinical characteristics of patients with pulmonary hypertension stratified by drug exposure are summarized in Table 9. In contrast to PDE5i and ERA, the odds of CCB use versus no CCB use is greater among patients with ADRD than those without ADRD (Table 10).

**Table 9:** Characteristics of patients included in the study with pulmonary hypertension stratified by history of phosphodiesterase-5 inhibitor (PDE5i) and calcium channel blocker (CCB) use.

Characteristic^1^	PDE5i only	PDE5i + CCB	CCB only	Neither
	n = 367	n = 521	n = 4,146	n = 2,772
**Demographics**				
Age, mean (SD)	75 (7)	76 (7)	79 (8)	78 (8)
Male	255 (69.5)	332 (63.7)	1,420 (34.2)	1,133 (40.9)
White	308 (83.9)	413 (79.3)	3,382 (81.6)	2,395 (86.4)
**Dementia risk factors**				
Smoking history				
Current smoker	27 (7.4)	38 (7.2)	336 (8.1)	237 (8.5)
Never smoker	143 (39.0)	228 (43.8)	1,992 (48.0)	1,296 (46.8)
BMI ≥ 25	314 (85.6)	469 (90.0)	3,461 (83.5)	2,227 (80.3)
Hypertension	328 (89.4)	507 (97.3)	4,024 (97.1)	2,485 (89.6)
Diabetes	160 (43.6)	259 (49.7)	2,000 (48.2)	1,116 (40.3)
Coronary artery disease	233 (63.5)	364 (69.9)	2,705 (65.2)	1,714 (61.8)
Ischemic stroke	50 (13.6)	106 (20.3)	979 (23.6)	478 (17.2)
Atrial fibrillation	192 (52.3)	319 (61.2)	2,518 (60.7)	1,327 (47.9)
Venous or pulmonary embolism	53 (14.4)	76 (14.6)	607 (14.6)	392 (14.1)
**ADRD**	19 (5.2)	49 (9.4)	684 (16.5)	370 (13.3)

^1^Reported as n (%), unless otherwise specified

Additional abbreviations: BMI, body mass index; ADRD, Alzheimer’s disease and related dementias.

**Table 10:** Odds ratio among patients with pulmonary hypertension for history of calcium channel blocker (CCB; amlodipine, nifedipine, or diltiazem) use in patients with Alzheimer’s disease and related dementias (ADRD) versus controls without ADRD. Fisher’s exact test for significance of odds ratios v. unity.

Analysis	Odds ratio (95% CI)	p-value
CCB v. no CCB	1.307 (1.146–1.492)	< 0.001

**Fig 1:**
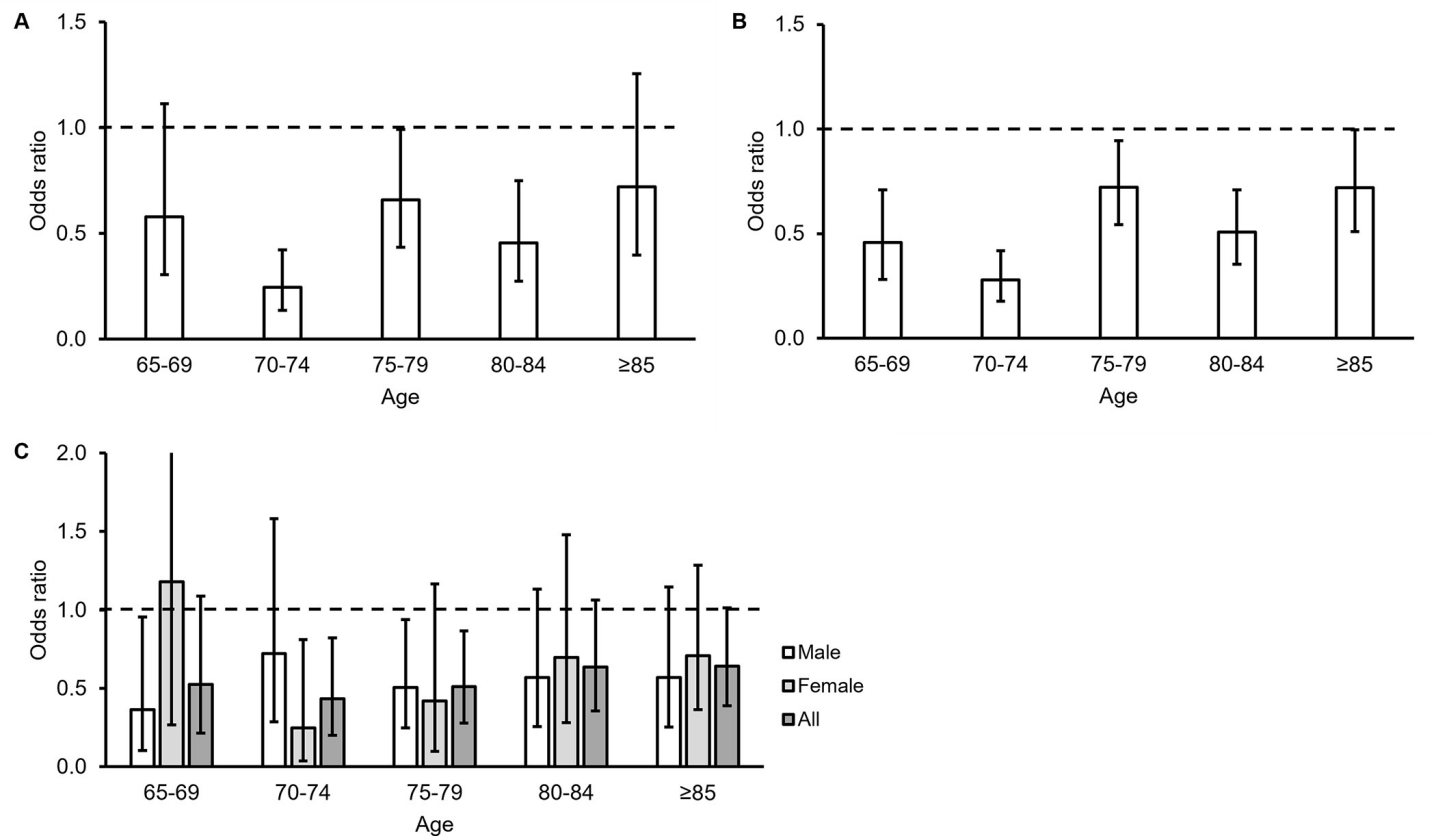
Odds ratios stratified by age among patients with (A) erectile dysfunction (ED), (B) benign prostatic hyperplasia (BPH), and C) pulmonary hypertension (pHTN) for history of phosphodiesterase-5 inhibitor use in patients with Alzheimer’s disease and related dementias (ADRD) versus controls without ADRD. Only males were included in calculations for ED and BPH. Error bars represent 95% confidence intervals.

Finally, we evaluated whether age modifies the association between history of PDE5i use and ADRD. We observed either statistical significance or a trend toward an odds ratio less than unity among males and females for each PDE5i indication tested with each five-year age group over 65 years, except for females with pHTN in the 65- to 69-years-old group (Fig 1).

## Discussion

We undertook the present study to reconcile the diametrically opposed conclusions of Fang *et al*. and Desai *et al*. regarding the protective effect of PDE5i on AD risk and to determine whether further observational studies and/or clinical trials to evaluate this association are warranted. We began by repeating the main epidemiologic investigations from those studies. Similar to the protective effect of PDE5i reported by Fang *et al*., we observed lower odds of PDE5i use in patients with AD and ADRD than in controls without ADRD among populations with ED, BPH, and pHTN. Congruent with the results of Desai *et al*., we observed similar odds ratios of PDE5i use and ADRD and ERA use and ADRD, among patients with pHTN. A major difference between the study of Desai *et al*. and our study is that their study classified patients taking ERA as the unexposed (control) group, while we classified patients without a history of use of the drug in question (PDE5i or ERA) as the unexposed group. Based on this comparison, they concluded that PDE5i do not decrease the risk of ADRD. An alternate explanation of their data could be that both PDE5i and ERA similarly reduce the risk of ADRD in patients with pHTN when compared with patients with untreated pHTN. Our data support the latter interpretation.

It therefore is likely that the disparity in the conclusions reported by Fang *et al*. and Desai *et al*. arises not from flaws in the experimental design chosen by Fang *et al*. but rather because the authors of the two studies asked different epidemiologic questions. The question asked by Fang *et al*. is whether treatment with sildenafil is associated with a lower risk of developing Alzheimer’s disease among diverse patient populations. The question asked by Desai *et al*. is whether treatment with PDE5i is associated with a lower risk of ADRD inherent to the drug class itself and independent of the effects of treating an underlying comorbidity. The answer to the latter question, according to the authors of that report, seems to be “no”, suggesting that the effect may be due to treatment of the underlying comorbidity. We approached the question posed by Desai *et al*. from a different angle, by evaluating patients with three different PDE5i indications and comparing treated patients to untreated control patients within each population. The consistent findings among patients with ED, BPH, and pHTN, along with the inverse association found with CCB in patients with pHTN, support a class effect of PDE5i. Furthermore, expanding on the findings of Desai *et al*., ERA also may be effective in reducing the incidence of ADRD among patients with pHTN.

Our work adds new and important findings to the literature. First, the observed association of PDE5i and ADRD risk is found in pHTN patients regardless of legal sex. In both males and females with pHTN, we observed lower odds of PDE5i use in patients with ADRD than in controls without ADRD. Our comparison of males and females was limited to patients with pHTN because this is the only FDA-approved indication for PDE5i for both sexes. Desai *et al*. included sex as a matching variable and did not address it further. Fang *et al*. compared hazard ratios of males and females separately and observed that while males had a hazard ratio less than unity (HR = 0.27, 95% CI: 0.21–0.34), females trended toward a hazard ratio less than unity but did not reach statistical significance (0.65, 95% CI: 0.41–1.02). They cited a lack of statistical power to explain their lack of statistical significance. Furthermore, they hypothesized that the difference between the sexes was dose-related. The average man in their study was taking a higher dose of sildenafil (mean = 75.9; SD = 32.4 mg) to treat ED, whereas the average woman in their study was taking a lower dose (mean = 22.1 mg, SD = 15.0 mg) to treat pHTN. If the effect size was due to dosing differences, one would expect it to disappear in males and females with pHTN, since one would expect their dosing regimens to be similar, with a recommended dose of 20 mg three times per day to 80 mg three times per day for both males and females [45]. However, we observed similar odds ratios for both males and females.

Second, on examining the question of dose and frequency more closely, we found, as did Fang *et al*., that the odds of PDE5i use is lower among patients with ADRD than those without ADRD for a wide range of PDE5i doses and frequencies. A different regimen is prescribed for sildenafil and tadalafil for each of their indications. When sildenafil is used to treat ED, the usual dose is 25 to 100 mg daily, as needed; however, when it is used to treat pHTN, the usual dose is 20 to 80 mg three times daily [45]. When tadalafil is used to treat ED, the usual dose is 2.5 to 5 mg daily; for BPH, the usual dose is 5 mg daily; however, when it is used to treat pHTN, the usual dose is 40 mg daily [44]. Regardless of the different indications with their widely varying recommended dosages and frequencies, we found decreased odds ratios across our study. This suggests that in humans, as in mice, the clinical effects of sildenafil and tadalafil, lipid-soluble drugs that cross the blood-brain barrier, may last far longer than their various plasma half-lives [26, 34, 47]. However, future studies using patient level data are required to determine the minimum dose necessary for these drugs to have maximal benefit in ADRD risk reduction.

Third, we observed similar odds ratios using either the more selective outcome of AD or the more inclusive outcome of ADRD for patients with ED, BPH, and pHTN. We also observed similar demographic and clinical characteristics between the AD and ADRD groups when looking at all patients with a PDE5i indication as well as only those with pHTN. Whether the similarities between the odds ratios for AD and ADRD are due to imperfect classification of dementia etiology in the medical record or a common mechanism whereby PDE5i and ERA reduce the risk of the various causes of ADRD remains unknown. In addition, the effects sizes we observed are consistent across all three FDA-approved indications for PDE5i we evaluated, decreasing the chance of confounding variables as a reason for our findings. Together, these novel findings support the use of more inclusive criteria for future studies, facilitating the availability of larger sample sizes for observational studies and randomized clinical trials.

Our results emphasize the importance of blood-brain-barrier permeability and the site of action of drugs in protecting against ADRD. Sildenafil and tadalafil may have multiple sites of action at which they work to decrease ADRD risk. Both drugs, as well as the less commonly prescribed PDE5i vardenafil, can cross the blood-brain barrier and access brain parenchyma and cerebral vascular smooth muscle cells directly, in addition to exerting effects in the endothelium [34, 47–49]. While we were unable to find a study evaluating the extent to which ERA cross the blood-brain barrier in humans, macitentan, an ERA for which we observed an odds ratio less than unity, is highly soluble in octanol, with a partition coefficient of 800:1 [50, 51]. Furthermore, ERA act on endothelial cells, which lie proximal to the blood-brain barrier.

In contrast to the studies by Fang *et al*. and Desai *et al*., we used an electronic medical record database (EMR) instead of insurance databases. The use of an EMR database for an entire health care system housing more than 1.4 million patients enables us to capture a patient population with diverse demographics that more accurately reflect Arkansas’ population. This includes patients with government funded insurance, privately funded insurance, and no insurance at all. In addition, the use of EMR allows us to follow up with chart review in future studies to more accurately assess the relationship between exposures and outcomes.

Several mechanisms have been hypothesized to explain the protective effect of PDE5i from ADRD in murine models and humans: increased cerebral blood flow, neuroprotection, and increased neurogenesis [19]. ERA may offer a protective effect from ADRD via increased cerebral blood flow and neuroprotection. Human cerebral arteries have been found to constrict upon stimulation by endothelin-1 (ET-1), the predominant endothelin in the cardiovascular system [52, 53]. This constriction is the net effect of agonism at endothelin receptor A (ET_A_), which mediates constriction, and endothelin receptor B (ET_B_), which mediates dilation [54]. ET-1 also mediates a pro-thrombotic state by increasing platelet aggregation, which may further exacerbate cerebral blood flow deficits [55]. ET-1 is also a pro-inflammatory cytokine, which may lead to direct neuronal damage via increased formation of reactive oxygen species [55]. Thus, there is mechanistic rationale to explain how ERA might protect against ADRD [51]. The precise mechanisms behind a protective effect of PDE5i and ERA on ADRD in humans, however, remain to be determined.

Our study has several important limitations. First, due to the study’s use of aggregate data without accessing individual patient charts, certain important factors could not be assessed: the temporal relationship between exposure and outcome, duration of treatment, dosages and frequency of drug administration, the specific etiology of each patient’s dementia, including genetic markers for high AD risk and family history, age of dementia onset, patient ethnicity, socioeconomic status, and educational attainment. The results of the present study will be used in the design of a subsequent study that will incorporate chart review to address each of these factors. Establishing the temporal relationship between exposure and outcome is particularly important. However, knowing the temporal relationship only will recategorize patients in the ADRD group from exposed to unexposed in the event the AD or ADRD diagnosis occurred prior to the patient taking the PDE5i or ERA, rendering any observed protective effect even more pronounced.

Second, as this was a retrospective study, randomization was not possible. Furthermore, without accessing individual patient charts, matching on multiple variables was impractical. In this case, restricting patients by indication is the most appropriate method of limiting confounding variables. Indeed, lack of restriction to a specific indication was the most important critique of the Fang *et al*. study by Desai *et al*. When we limited eligible patients to ED, BPH, and pHTN, the three FDA-approved indications for PDE5i, and we observed similar effects within each group.

Third, while Baptist Health’s EMR database hosts records for more than 1.4 million patients, it is limited in geography. Fourth, our sample sizes for individual ERA limited our ability to complete further analyses of two of these drugs. This is an opportunity for collaboration with researchers at other institutions. Fifth, our study did not seek to determine the biological mechanisms responsible for the decreased odds of PDE5i among ADRD patients in this study.

Sixth, as with the studies completed by Fang *et al*. and Desai *et al*., our study assesses the potential for using PDE5i inhibitors as a preventive medication, not as a treatment to slow progression of mild cognitive impairment or ADRD after the diagnosis has been made. A prospective phase III clinical trial will be required to assess definitively the efficacy of PDE5i for secondary prevention of ADRD. Again, this will be facilitated by the fact that PDE5i are known to be relatively safe drugs, are inexpensive and widely available, and are already approved for use by the FDA [56, 57].

As the world’s population continues to age, the prevalence of ADRD and the associated healthcare costs will continue to increase in the absence of effective strategies to prevent these dementias [58]. It is therefore important to find new therapies to prevent and treat ADRD. In this study, we have demonstrated a decreased odds of PDE5i use in patients with ADRD versus controls without ADRD in all three FDA approved indications for PDE5i. This finding is consistent in both males and females, with either sildenafil or tadalafil, and among patients with a diagnosis of AD-related dementias, not just Alzheimer’s disease proper. These observations will facilitate the use of larger sample sizes in future retrospective studies and clinical trials and may also increase the number of patients for whom these therapies may be indicated.

## Supporting information

S1 Table Slicer Dicer search terms. (DOCX)

S2 Table Patient frequencies used in calculation of odds ratios. (DOCX)

S3 Table Patient frequencies, odds ratios, and 95% confidence intervals from Fig 1. (DOCX)
