# Supplementary material for: A case-control study of phosphodiesterase-5 inhibitor use and Alzheimer’s disease and related dementias among male and female patients aged 65 years and older supporting the need for a phase III clinical trial
Source: PLoS One. 2023 Oct 18;18(10):e0292863. doi: 10.1371/journal.pone.0292863 (PMC10584171; doi:10.1371/journal.pone.0292863)
Supplement: S1 Table — (DOCX) [file pone.0292863.s001.docx]

**S1 Table. Slicer Dicer search terms.**

| **Diagnosis** | **Search terms** |
| --- | --- |
|  |  |
| Alzheimer’s disease and related dementias | ICD-10: G30.*, F01.*, F02.*, F03.*  SNOMED: Alzheimer’s disease  EDG concept dementia |
| Alzheimer’s disease | ICD-10: G30.* |
| Erectile dysfunction | ICD-10: N52.*  EDG concept erectile dysfunction |
| Benign prostatic hyperplasia | ICD-10: N40.*  SNOMED: benign prostatic hyperplasia |
| Pulmonary hypertension | ICD-10: I27.0*, I27.2* |
| Hypertension | ICD-10: I10.*, I15.* |
| Diabetes | ICD-10: E08.*, E10.*, E11.* |
| Coronary artery disease | ICD-10: I25.* |
| Ischemic stroke | ICD-10: I63.* |
| Atrial fibrillation | ICD-10: I48.* |
| Venous embolism | ICD-10: I82.*, I26.* |
| Current smoker | Heavy smoker, Light smoker, Some days, Everyday |
