# Supplementary material for: A case-control study of phosphodiesterase-5 inhibitor use and Alzheimer’s disease and related dementias among male and female patients aged 65 years and older supporting the need for a phase III clinical trial
Source: PLoS One. 2023 Oct 18;18(10):e0292863. doi: 10.1371/journal.pone.0292863 (PMC10584171; doi:10.1371/journal.pone.0292863)
Supplement: S2 Table — (DOCX) [file pone.0292863.s002.docx]

**S2 Table. Patient frequencies used in calculation of odds ratios.**

| Table | Analysis | Population | Cases | | Controls | | |
| --- | --- | --- | --- | --- | --- | --- | --- |
|  |  |  | Exposed | Unexposed | Exposed | Unexposed |  |
| 2 | PDE5i v. no PDE5i | ED | 112 | 210 | 3,965 | 2,663 |  |
|  |  | BPH only | 96 | 2,258 | 1,603 | 16,680 |  |
|  |  | pHTN | 68 | 1,057 | 822 | 5,871 |  |
|  | ERA v. no ERA | pHTN | 9 | 1,116 | 146 | 6,547 |  |
| 4 | PDE5i v. no PDE5i | ED | 41 | 81 | 3,965 | 2,663 |  |
|  |  | BPH only | 47 | 881 | 1,603 | 16,680 |  |
|  |  | pHTN | 24 | 317 | 822 | 5,871 |  |
|  | ERA v. no ERA | pHTN | 1 | 340 | 146 | 6,547 |  |
| 6 | PDE5i v. no PDE5i | Male | 40 | 385 | 549 | 2,172 |  |
|  |  | Female | 28 | 672 | 273 | 3,699 |  |
|  | ERA v. no ERA | Male | 3 | 422 | 40 | 2,681 |  |
|  |  | Female | 6 | 694 | 106 | 3,866 |  |
| 7 | Sildenafil only v. no PDE5i | ED | 78 | 231 | 2,538 | 2,795 |  |
|  | Tadalafil only v. no PDE5i |  | 24 | 231 | 906 | 2,795 |  |
|  | Sildenafil only v. no PDE5i | pHTN | 55 | 1,059 | 652 | 5,877 |  |
|  | Tadalafil only v. no PDE5i |  | 9 | 1,059 | 106 | 5,877 |  |
| 8 | Macitentan v. no macitentan | pHTN | 6 | 1,120 | 107 | 6,588 |  |
|  | Bosentan v. no bosentan |  | 2 | 1,124 | 18 | 6,677 |  |
|  | Ambrisentan v. no ambrisentan |  | 1 | 1,125 | 36 | 6,659 |  |
| 10 | CCB v. no CCB | pHTN | 734 | 392 | 3,943 | 2,752 |  |
|  | PDE5i v. no PDE5i | pHTN only | 13 | 178 | 224 | 1,324 |  |
