# Supplementary material for: A case-control study of phosphodiesterase-5 inhibitor use and Alzheimer’s disease and related dementias among male and female patients aged 65 years and older supporting the need for a phase III clinical trial
Source: PLoS One. 2023 Oct 18;18(10):e0292863. doi: 10.1371/journal.pone.0292863 (PMC10584171; doi:10.1371/journal.pone.0292863)
Supplement: S3 Table — (DOCX) [file pone.0292863.s003.docx]

**S3 Table. Patient frequencies, odds ratios, and 95% confidence intervals from Figure 1.**

| Population | Age range | Sex | Cases | | Controls | | OR (95% CI) |
| --- | --- | --- | --- | --- | --- | --- | --- |
|  |  |  | Exposed | Unexposed | Exposed | Unexposed |  |
| ED | 65-69 | Male | 22 | 17 | 1673 | 748 | 0.578 (0.305 – 1.114) |
|  | 70-74 |  | 18 | 43 | 1298 | 756 | 0.245 (0.137 – 0.422) |
|  | 75-79 |  | 42 | 58 | 737 | 670 | 0.659 (0.434 – 0.992) |
|  | 80-84 |  | 22 | 58 | 338 | 404 | 0.456 (0.274 – 0.750) |
|  | ≥85 |  | 19 | 56 | 107 | 226 | 0.721 (0.398 – 1.256) |
| BPH | 65-69 | Male | 21 | 148 | 1226 | 3929 | 0.458 (0.280 – 0.710) |
|  | 70-74 |  | 23 | 299 | 1211 | 4355 | 0.278 (0.176 – 0.418) |
|  | 75-79 |  | 62 | 465 | 812 | 4387 | 0.722 (0.543 – 0.944) |
|  | 80-84 |  | 37 | 595 | 402 | 3269 | 0.508 (0.353 – 0.709) |
|  | ≥85 |  | 46 | 1096 | 169 | 2890 | 0.720 (0.510 – 0.996) |
| pHTN | 65-69 | Male | 4 | 28 | 154 | 380 | 0.365 (0.104 – 0.954) |
|  |  | Female | 3 | 40 | 39 | 587 | 1.179 (0.266 – 3.463) |
|  |  | All | 7 | 68 | 193 | 967 | 0.526 (0.215 – 1.089) |
|  | 70-74 | Male | 7 | 34 | 137 | 473 | 0.723 (0.286 – 1.582) |
|  |  | Female | 2 | 88 | 72 | 735 | 0.249 (0.038 – 0.812) |
|  |  | All | 9 | 122 | 209 | 1208 | 0.433 (0.201 – 0.821) |
|  | 75-79 | Male | 11 | 76 | 151 | 520 | 0.505 (0.247 – 0.938) |
|  |  | Female | 3 | 97 | 63 | 817 | 0.421 (0.098 – 1.165) |
|  |  | All | 14 | 173 | 214 | 1337 | 0.510 (0.278 – 0.867) |
|  | 80-84 | Male | 9 | 94 | 66 | 387 | 0.570 (0.255 – 1.133) |
|  |  | Female | 7 | 138 | 50 | 674 | 0.697 (0.281 – 1.478) |
|  |  | All | 16 | 232 | 116 | 1061 | 0.636 (0.356 – 1.064) |
|  | ≥85 | Male | 9 | 156 | 43 | 418 | 0.569 (0.253 – 1.146) |
|  |  | Female | 13 | 336 | 50 | 907 | 0.708 (0.363 – 1.284) |
|  |  | All | 22 | 492 | 93 | 1325 | 0.641 (0.388 – 1. 013) |
